# Homeobox 27, a Homeodomain Transcription Factor, Confers Tolerances to CMV by Associating with Cucumber Mosaic Virus 2b Protein

**DOI:** 10.3390/pathogens11070788

**Published:** 2022-07-12

**Authors:** Usha Kumari Rattan, Surender Kumar, Reenu Kumari, Monika Bharti, Vipin Hallan

**Affiliations:** 1 Plant Virology Lab, CSIR-Institute of Himalayan Bioresource Technology, Palampur 176061, India; rattan.usha77@gmail.com (U.K.R.); nishu.rana1@gmail.com (S.K.); rkaundal30@gmail.com (R.K.); monikabhagat19@gmail.com (M.B.); 2 Academy of Scientific and Innovative Research (AcSIR), Ghaziabad 201002, India; 3 College of Horticulture and Forestry, Dr. Y. S. Parmar University of Horticulture and Forestry, Thunag, Mandi 175048, India

**Keywords:** Cucumber mosaic virus, CMV, 2b protein, plant–virus interaction, protein–protein interaction, transcription factor

## Abstract

Transcription factors (TFs) play an important role in plant development; however, their role during viral infection largely remains unknown. The present study was designed to uncover the role transcription factors play in Cucumber mosaic virus (CMV) infection. During the screening of an *Arabidopsis thaliana* (Col-0) transcription factor library, using the CMV 2b protein as bait in the yeast two-hybrid system, the 2b protein interacted with Homeobox protein 27 (HB27). HB27 belongs to the zinc finger homeodomain family and is known to have a regulatory role in flower development, and responses to biotic and abiotic stress. The interaction between CMV 2b and HB27 proteins was further validated using in planta (bimolecular fluorescence complementation assay) and in vitro far-Western blotting (FWB) methods. In the bimolecular fluorescence complementation assay, these proteins reconstituted YFP fluorescence in the nucleus and the cytoplasmic region as small fluorescent dots. In FWB, positive interaction was detected using bait anti-MYC antibody on the target HB27-HA protein. During CMV infection, upregulation (~3-fold) of the HB27 transcript was observed at 14 days post-infection (dpi) in *A. thaliana* plants, and expression declined to the same as healthy plants at 21 dpi. To understand the role of the HB27 protein during CMV infection, virus accumulation was determined in HB27-overexpressing (HB27 OE) and knockout mutants. In HB27-overexpressing lines, infected plants developed mild symptoms, accumulating a lower virus titer at 21 dpi compared to wild-type plants. Additionally, knockout HB27 mutants had more severe symptoms and a higher viral accumulation than wild-type plants. These results indicate that HB27 plays an important role in the regulation of plant defense against plant virus infection.

## 1. Introduction

Transcription factors (TFs) are key regulators in many developmental processes in plants. TFs have unique DNA-binding domains, which bind to the regulatory elements present in promoter regions and lead to the activation or repression of the target gene [1,2]. Approximately, 5–10% of *Arabidopsis thaliana* genome encodes for TFs and more than two thousand (2000) have been identified [3,4]. Homeobox is an important TF family and in Arabidopsis approximately hundred (100) homeobox genes have been identified, which are classified into six groups: ZF-HD (zinc finger homeodomain), HD-Zip (homeodomain-leucine zipper), Bell (named after the well-known Bell domain), Wuschel-related homeobox (WOX), Knotted1-like homeobox (KNOX) and PHD finger (plant homeodomain associated with a finger domain) [4,5,6].

The ZF-HD subfamily has fourteen members in Arabidopsis [7]. Plant-specific ZF-HD TFs are important for plant growth, development and stress responses [7,8]. Members of the ZF-HD subfamily contain only exons and a conserved region containing His and Cys residues [2,7]. ZF-HD proteins contain two domains: an HD (homeodomain) and a ZF domain of the C_2_H_2_ type, which binds both DNA and protein–protein complexes for modulation of target gene expression [2,9,10,11]. Proteins with the ZF-HD domain typically form homodimers and heterodimers with specific DNA sequences with a consensus signal of ATTA [7]. Overexpression of AtZHD1 promotes drought tolerance in Arabidopsis through binding to the promoter region of the early response to dehydration stress 1 gene [8]. In soybean, the homeodomain of GmZF-HD1 and GmZF-HD2 binds to the ATTA-rich region in the promoter of the *calmodulinisoform 4* gene and this binding upregulates the expression of the pathogen-responsive *calmodulinisoform 4* gene [12,13]. A ZHD was found to act as a potential regulator of a gene encoding phosphoenolpyruvate carboxylase in *Flaveria trinervia* [2]. Similarly, HD-ZIP subfamily members regulate the abiotic stress responses in plants. In rice, HD-ZIP homeobox genes were upregulated at various stages during abiotic stress [14,15]. AtHB12 and AtHB7 are candidate Arabidopsis genes that play roles in water stress responses [16]. These studies showed that TFs play important roles in biotic and abiotic stress, but very little information was available on their response during virus infection.

For the successful establishment of infection, viral proteins associate with multiple host factors and redirect these factors for their replication, translation, suppression of the host defense and their local and systemic movement in plants [17,18,19,20,21]. Cucumber mosaic virus (CMV) is an important virus with a very wide host range, infecting both monocots and dicots [22,23]. The CMV genome contains three genomic and two subgenomic positive sense RNAs, designated RNA1, RNA2, RNA3, RNA4 and RNA4A, respectively [23]. RNA 1 and RNA 2 encode for the 1a and 2a proteins; these proteins form a replicase complex, which helps in the replication of viral genome [24,25]. RNA 3 and RNA 4 encode the movement protein and coat protein which, respectively, are essential in virus movement in the host and encapsidate the genomic RNAs [26,27]. RNA 4a encodes the 2b protein, which functions as a viral suppressor of RNA silencing and also helps in the systemic movement of the virus [17]. The CMV 2b interacts with host RNA silencing components and disrupts the expression of microRNA-regulated genes and DNA methylation [28]. The 2b protein-expressing transgenic A. thaliana plants exhibit a genome-wide decrease in CHH and CHG methylation in their transposable elements and coding genes [29]. The silencing suppressor targets the AGO1 and AGO4 proteins, by binding directly with their PIWI and PAZ domains and reducing the slicing action [28,30]. Additionally, 2b protein interacts with 21–24 nt siRNA and long 55 nt dsRNA duplexes and inhibits host RNA silencing components [31]. The 2b protein interacts with Arabidopsis catalase 3 to reduce catalase activity, specifically the removal of cellular hydrogen peroxide, which promotes the growth of necrotic symptoms after CMV infection [32]. The 2b protein is composed of distinct domains with specific roles: the region containing amino acids 1–61 contains nuclear localization signals (NLS), a region that binds dsRNA/siRNA, and phosphorylation sites; the region containing amino acids 38–110 binds AGO proteins; and the region containing amino acids 62–110 lacks AGO-binding affinity [19]. The advent of viral symptoms will be due to direct molecular interaction between a viral protein and a host factor such as a transcription factor [33]. The direct association of 2b protein with cucumber RDR1s suppresses RDR1-mediated host defense in cucumber plants [19]. These studies showed that CMV 2b is a multifunctional protein and performs various functions for the establishment of infection. In this study, we identified that the HB27 TF interacts with the CMV 2b protein. Homeobox genes are reported to have roles mainly in plant development and abiotic stress. The functional analysis of the interaction between TFs and viral proteins is essential for the elucidation of the role of these transcriptional regulators in the modulation of defense.

## 2. Material and Methods

### 2.1. Plant Material and Growth Conditions

*Arabidopsis thaliana* ecotype Columbia-0 (Col-0) seedlings were grown for 2 weeks on half-strength Murashige and Skoog (MS) plates before being transplanted into a cocopeat: vermiculite: perlite (1:1:1) mixture for 4 weeks at 22 °C, humidity (60%) in 16/8 h photoperiod (150 mmol m^–2^ s^–1^ fluorescent white light) [34]. *Nicotiana benthamiana* plants were grown on soil for 3–4 weeks for the bimolecular fluorescence complementation (BiFC) assay experiment in the plant growth chamber (temperature 22 ± 2 °C with 60% humidity).

### 2.2. Construct Preparation

For yeast two-hybrid (Y2H) analysis, the coding sequence of the *2b* gene of CMV subgroup II (GenBank accession no. FR821516) was cloned into the pGBKT7 vector, which has restriction sites for *Nde*I and *Sma*I, named 2b-pGBKT7. Similarly, the restriction sites *Xho*I and *Bgl*II were used to clone HB27 (GenBank accession no. LN852659.1) in the pGADT7 vector and designated as HB27-pGADT7. The Arabidopsis "transcription factor only library” (a gift from Prof. Nobutaka Mitsuda, AIST, Ibaraki, Japan) was received in the pDEST-GAD424 prey vector. For the BiFC assay, HB27 protein coding sequence was cloned in a pSPYNE(R)173 vector with YFP-N-terminus at *Xho*I and *Spe*I restriction sites, while the 2b coding sequence was cloned into a pSPYCE(MR) vector with YFP-C-terminus at *BamH*I and *Xho*I restriction sites, termed HB27-pSPYNE(R)173 and 2b-pSPYCE(MR), respectively. For heterologous expression (required for far-Western blotting; FWB), forward primers with HA- and MYC-tags were incorporated for cloning of HB27 and CMV 2b, respectively, into the pET28 vector. The 2b coding sequence was cloned into the pET28a vector at *BamH*I and *Xho*I sites and named CMV2b-MYC-pET28a, while HB27 was cloned into a pET28a vector at *Nde*I and *Xho*I restriction sites, and named HB27-HA-pET28a. For overexpression of HB27 in Arabidopsis, the gene was cloned into pCAMBIA-1302 vector (as a fusion protein with GFP) at restriction sites *Spe*I and *Bgl*II, and named HB27-pCAMBIA-1302. All recombinant plasmids were selected using colony PCR and validated using Sanger sequencing. Details of primers used in this study were given in Supplementary Table S1.

### 2.3. Yeast Two-Hybrid Screening

Protein–protein interaction was carried out using the Y2H system (Clontech, TakaraBio, Kusatsu, Japan). CMV 2b protein (FR821516) was used as bait (in pGBKT7 vector) for screening of the Arabidopsis TF library (pDEST-GAD424 vector) as prey for co-transformation in yeast *Saccharomyces cerevisiae* strain AH109. The screening was performed as per [26]. Confirmation of interaction was carried out on SD/-Leu/-Trp/-His/-Ade (SD/-LTHA) dropout media containing 4mM AT and using β-X-gal assay. The identity of the interactors was confirmed using colony PCR, followed by sequencing. One-to-one interaction between HB27 and 2b was also confirmed using the GAL4-based Y2H System (Clontech). For this, HB27-pGADT7 and 2b-pGBKT7 were transformed in yeast *S. cerevisiae* strain Y187 and Y2H. Yeast transformants were screened on minimal medium plates SD/-LTHA at 30 °C for 5–7 days. Positive transformants were further screened on /SD-Leu/-Trp plates for X-gal assay.

### 2.4. Bimolecular Fluorescence Complementation (BiFC) Assay

BiFC assay was performed as detailed previously [26], using pSPYNE173 and pSPYCE (MR) vectors described [35]. The recombinant plasmids 2b-pSPYNE173 and HB27-pSPYCE were transformed in *Agrobacterium tumefaciens* GV3101 strain using the freeze–thaw method [36]. Agroinfiltration was performed with a medium (10 mM MES, 10 mM MgCl_2_, pH 5.6 and acetosyringone 200 µM) at OD_600_ of 0.3 (Tombusvirus P19 protein, a VSR) and 0.5 (other constructs). Agroinfiltration was performed on 4–6-week-old *N. benthamiana* leaves. YFP fluorescent signals were observed 48 h after infiltration under a confocal microscope (LSM510Meta, Carl Zeiss, Jena, Germany), at 514 nm excitation and emission spectra at 530–600 nm.

### 2.5. Expression and Purification of HB27 and CMV 2b Proteins

For heterologous expression, HB27-HA-pet28a and CMV2b-MYC-pet28a constructs were transformed into *E. coli* strain BL21 (DE3). Expression of recombinant proteins was induced by 1mM IPTG at 37 °C for 3 h. Induced proteins were purified using Ni-NTA columns (Fast Start Kit; Qiagen, Hilden, Germany), as both contain His-tag. The concentration and purity of proteins were checked using Bradford assay and SDS-PAGE (12%), respectively.

### 2.6. Far-Western Blotting 

The protein–protein interaction was further validated using FWB, an in vitro protein–protein interaction confirmation method derived from Western blotting [37]. In FWB, purified CMV 2b-MYC protein was used as bait and HB27-HA as prey protein. Both proteins were run on SDS-PAGE (12% gel) and transferred to polyvinylidene difluoride membrane. Proteins on the membrane were denatured and renatured in AC buffer (100 mM NaCl, 20 mM Tris [pH 7.6], 0.5 mM EDTA, 10% glycerol, 0.1% Tween-20, 2% skimmed milk powder, 1 mM DTT, and urea) by gradually decreasing urea concentration from 6 M to 0 M at each incubation step as described by [37]. The membrane was blocked by incubating in 5% skimmed milk for 1 hour at room temperature, followed by washing three times with PBST. Then, the membrane was incubated with purified bait protein (5 µg) in binding buffer (AC buffer without urea) overnight at 4 °C. The first detection was by anti-MYC antibody followed by anti-HA antibody. Bovine serum albumin was used as a negative control.

### 2.7. Generation of HB27 Overexpressing and Knockout Lines in Arabidopsis

For the generation of HB27 stable overexpressing Arabidopsis lines (HB27 OE), recombinant HB27-pCAMBIA 1302 was transformed into the GV3101 strain of agrobacterium. Arabidopsis ecotype Columbia-0 (Col-0) plants were grown under optimum conditions (as above). The vacuum infiltration method was used for agro-transformation. Transformed seeds, i.e., T0 seeds were screened on MS medium with 25 mg/mL hygromycin (selection marker) plates. Hygromycin-resistant plants were transferred to pots and checked for integration of T-DNA using hygromycin- and GFP-specific primers. Seeds collected from positive plants were grown in selection conditions up to T3 generation and were used for further experiments. The knockout lines of HB27 (*hb27 KO*) were obtained from the Arabidopsis Biological Resource Center (ABRC, SALK id: SALK_092897C). The mutant seeds were screened on kanamycin selection (20 mg/L) plates. The T-DNA insertion and homozygosity were confirmed through universal left-border primer LBb1.3 and gene-specific full-length primers of HB27. The HB27 expression in HB27 OE and *hb27 KO* lines was checked in transcripts using qRT-PCR and protein expression using Western blotting. In qRT-PCR, the host 18S rRNA gene was used as an internal control for normalization [38,39].

### 2.8. Virus Inoculation, Sample Collection, RNA Extraction

Mechanical inoculation of the CMV-SG isolate was performed using 100 mM phosphate buffer (pH 8.0) as described by [40]. Samples were collected in liquid N_2_ and immediately stored at −80 °C prior to RNA extraction. Total RNA was extracted and the transcript level of CMV coat protein was checked using qRT-PCR at different time intervals (7, 14 and 21 days post-infection; dpi) by RNA isolation kit (Thermo Scientific, Waltham, MA, USA).

### 2.9. Real-Time PCR

RNA isolation kit (Macherey-Nagel^TM^) was used to extract total RNA from healthy and CMV-inoculated cucumber leaf samples. For cDNA synthesis, 2 µg of total RNA was used and cDNA synthesis was performed using Verso cDNA synthesis kit (Thermo Scientific), following manufacturer’s instructions. Quantitative expression studies were performed in Real-Time PCR (Himedia Insta Q96, Maharashtra, India) using 2.0 µL of diluted cDNA (1:10), 5.0 µL 2× SYBR master mix in 10 µL reaction. The PCR reaction condition included 20 s at 95 °C, 40 cycles each of 15 s at 95 °C and 1 min at 58 °C. The 2^−ΔΔCt^ method was used to calculate relative expression. 18s rRNA was used as internal control, andthree biological replicate were taken for qRT-PCR analysis and each sample was a pool of three plants.

### 2.10. Protein Isolation and Western Blotting

Total protein was extracted from inoculated and healthy leaves at different time intervals with extraction buffer (7.5 mM Tris HCl, pH 6.8, 7.5% β-mercaptoethanol, 9 M urea). For detection, total proteins were fractionated on 12% SDS-PAGE gel and Western blotting was performed with specific primary antibody raised in rabbits in 1:5,000 dilutions. HRP conjugated secondary antibody in 1:10,000 dilutions (anti-rabbit or mice, from Sigma-Aldrich, St. Louis, MO, USA) was used. The signals were captured in a chemiluminescent detector (Azure Biosystems C300, Dublin, CA, USA) using an ECL substrate. Ponceau S stain was used to check the equal loading and transfer of proteins.

## 3. Results

### 3.1. HB27 Transcription Factor Interacts with CMV 2b Protein

**Figure 1:**
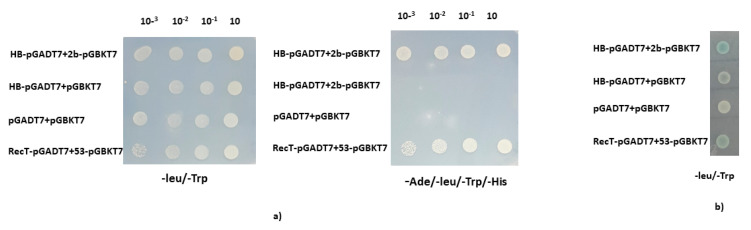
Cucumber mosaic virus 2b protein interacts with HB27 transcription factor, as demonstrated using a yeast two-hybrid experiment. (**a**) Dilution assay on nutritional selection dual (-LT) and quadruple dropout (-ALTH) media, to exhibit the growth of yeast transformants. Negative controls included HB27 protein with empty bait vector (pGBKT7), and both empty vectors (pGBKT7 and pGADT7) and positive controls includes the pGBKT7-p53 and pGADT7-T antigens. (**b**) In a galactosidase experiment, the blue color indicates a positive interaction of HB27 with 2b.

Transcription factors regulate the expression of target genes by interacting with the regulatory elements present in the promoter region [41]. CMV 2b protein is an important pathogenicity determinant, having multiple roles in the virus infection cycle. The primary goal of this study is to determine the involvement of transcription factors (TFs) in CMV pathogenesis or how virus suppressor protein modifies plant defense by binding the TFs and inhibiting the downstream expression of their target genes. The Arabidopsis TF library in the pDEST-GAD424 prey vector was screened using CMV 2b protein bait (pGBKT7-2b) in Y2H, to identify transcription factors that interact with the CMV-2b protein. A transcription factor, HB27, was found to interact with the CMV 2b protein. Direct interaction of HB27-pGADT7 with 2b-pGBKT7 was confirmed using dilution assay on quadruple-dropout selection medium plates (SD/-LTHA) supplemented with aureobasidin (0.2 µg/mL) (Figure 1). In the X-gal assay, blue color was observed during the HB27 and CMV 2b protein interaction. These results showed that HB27 directly associates with CMV2b protein in yeast cells.

### 3.2. Confirmation of Interaction Using in Planta and In Vitro Assays

**Figure 2:**
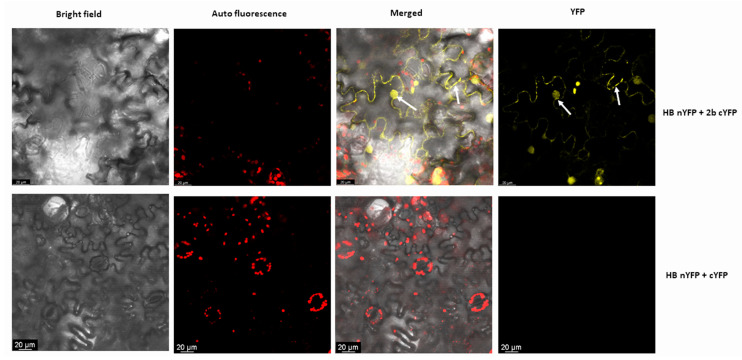
BiFC assay showing interaction between CMV2b and HB27 in *N. benthamiana* leaf epidermal cells under a confocal microscope. After 48 h, leaves infiltrated with HB27-nYFP and 2b-cYFP were studied under a confocal microscope, revealing fluorescent signals. Strong YFP fluorescence signals were observed in cytoplasm and nucleus, confirming interaction between HB27 and 2b proteins as indicated by arrows. No signals were observed in negative controls (HB27-nYFP and cYFP).

In BiFC, Hb27 was fused to the N-terminal half of YFP (HB-nYFP) and 2b protein was fused at the C-terminal half of YFP (2b-cYFP), respectively. Both fused proteins were infiltrated into the abaxial epidermis of *N. benthamiana* leaves along with the p19 suppressor protein. Forty-eight hours after infiltration, leaves were observed under a confocal microscope. Yellow fluorescence signals were observed as punctuate spots in the cytoplasm and in the nucleus (shown by white arrows), which confirmed the interactions between these proteins. No fluorescence signals were observed in the vector control (Figure 2).

**Figure 3:**
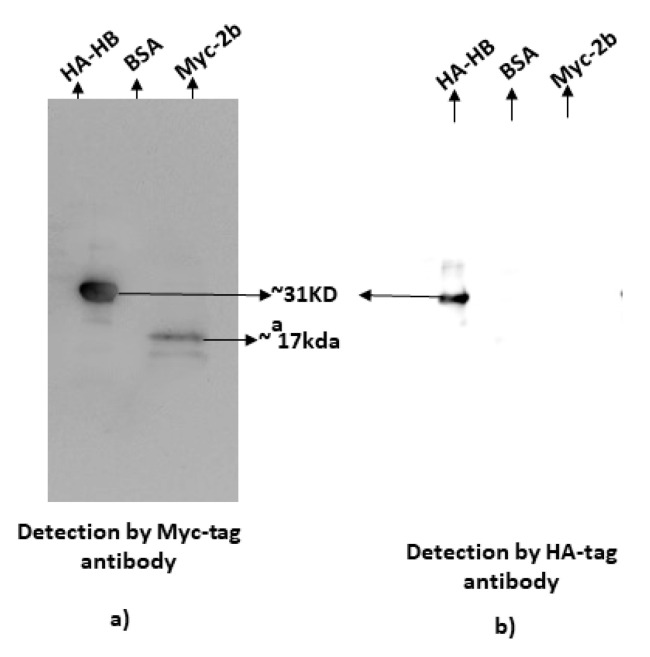
Far-Western blotting for confirmation of interaction between CMV-2b and HB27. HB27-HA-pET28a and CMV2b-MYC-pET28a proteins were purified, separated on SDS-PAGE, transferred to a polyvinylidene difluoride membrane and incubated with the bait protein (CMV2b-MYC-pET28a). (**a**) The membrane was treated with anti-MYC antibody to confirm the interaction between the proteins. (**b**) The same membrane was stripped and anti-HA antibody was used to detect HB27-HA fusion protein.

The association of CMV 2b with HB27 was also validated using far-Western blotting. In this method, HB27-HA was fixed to the membrane and then after blocking and treatment with 2b-MYC bait, the membrane was washed. Prey protein bound to bait protein was detected using anti-MYC antiserum. Positive signals for the binding of bait protein were observed only with HB27-HA and no signals were observed in the negative control, i.e., bovine serum albumin. 2b-MYC was also blotted to the membrane as a positive control for the detection of MYC-tagged protein (Figure 3a). The same membrane was stripped and re-probed with anti-HA antiserum. After re-probing, signals were observed only in HB27, confirming the presence of HB27 protein on the membrane (Figure 3b). HA-tagged Hb27 and MYC-tagged 2b proteins were expressed and purified from bacterial cells, and stained using Coomassie brilliant blue along with negative control bovine serum albumin (Supplementary Figure S1). Both in vivo and in vitro assays confirmed the interaction between CMV 2b and HB27.

### 3.3. Effect of CMV Infection on HB27 Expression

**Figure 4:**
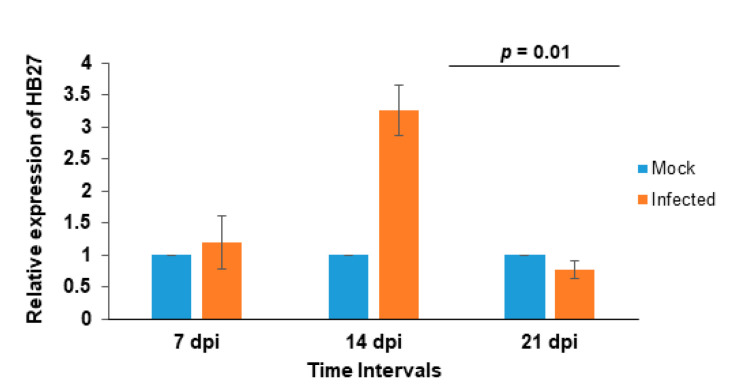
Effect of CMV infection on HB27 regulation. CMV was mechanically inoculated on Arabidopsis leaves and samples were collected at 7, 14 and 21 dpi. Transcript accumulation of HB27 was determined using qRT-PCR from three biological repetitions and the 18S rRNA gene used as an internal control to normalize the data. Relative accumulation of transcript was calculated using the 2^−ΔΔCt^ method. Blue bars represent mock samples and orange bars CMV-inoculated plants. Error bars represent the mean standard deviation ± SD (*n* = 3).

To understand the regulation of HB27 during viral infection, CMV was mechanically inoculated on the leaves of two-week-old Arabidopsis plants. Initial CMV symptoms were observed at 5 dpi, and systemic infection after 7 dpi. Samples were collected from virus-inoculated and healthy plants at 7, 14 and 21 dpi and analyzed using qRT-PCR. Initially, HB27 expression upregulation ~1.2- (at 7 dpi) to 3.2-fold (at 14 dpi) and at 21 dpi minor downregulation was observed in comparison to healthy plants (Figure 4). These results showed that CMV infection alters the expression of HB27.

### 3.4. Expression Analysis of HB27 in Overexpressing and Knockout Lines

**Figure 5:**
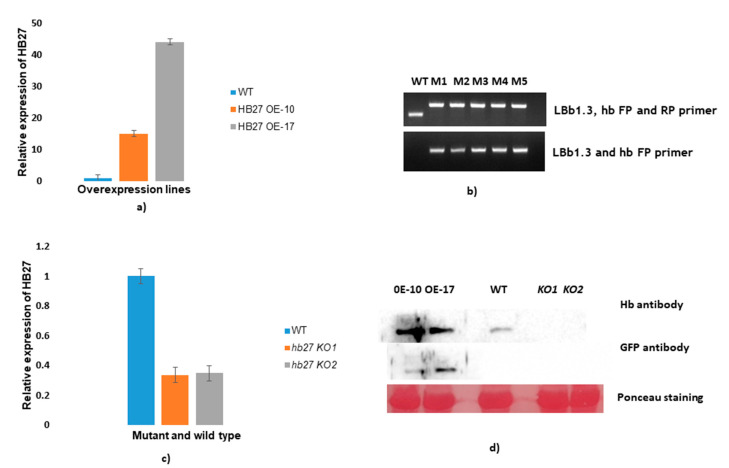
Generation of HB27 overexpression lines and analysis of knockout lines. Relative expression of HB27 was determined using qRT-PCR and compared with wild-type plants. (**a**) HB27 overexpressing lines were generated by transforming plants with HB27-pCAMBIA-1302 into Arabidopsis Col-0. (**b**) LBb1.3 (universal left-border primer) and hb27 (gene-specific HB27 full-length primers) were used to validate T-DNA insertion and homozygosity. (**c**) Relative expression of HB27 was determined in knockout lines. (**d**) HB27 and GFP tag antibodies were used to check the protein expression in both HB27 OE and *hb27-KO* lines compared to wild-type plants.

The role of HB27 during CMV infection was further investigated using Arabidopsis lines overexpressing HB27. Two lines (10 and 17) were selected for checking the expression of HB27 transcript by qRT-PCR and immunoblotting using anti-HB27 antiserum. The transcript level of HB27 was higher in overexpressing lines (~15–45-fold) when compared to wild-type Col-0 plants, determined using qRT-PCR (Figure 5a). In HB27 OE lines, there is early bud and flower development compared to wild-type plants. In addition, HB27 protein expression was higher in overexpressing lines than in wild-type plants (Figure 5d), determined by Western blotting using HB27 and GFP-specific antisera. HB27 knockout lines were genotyped, and 5 out of 40 plants were found to be homozygous for mutations in the HB27 gene. Full-length HB27 and LBb1.3 primers were used to evaluate T-DNA insertion in knockout lines through PCR (Figure 5b). Single band amplification in mutant plants reflected those plants that were homozygous for mutation. Two lines (named M1 and M2) were selected for checking the expression of HB27 transcript using qRT-PCR and immunoblotting using anti-HB27 antiserum. When compared to wild-type Col-0, the transcript level of HB27 in knockout *hb27 KO* lines (33% of wild type) was barely detectable (Figure 5c). Similarly, no protein expression was detected in knockout lines (Figure 5d).

### 3.5. Effect of HB27 Overexpression on CMV Pathogenesis

**Figure 6:**
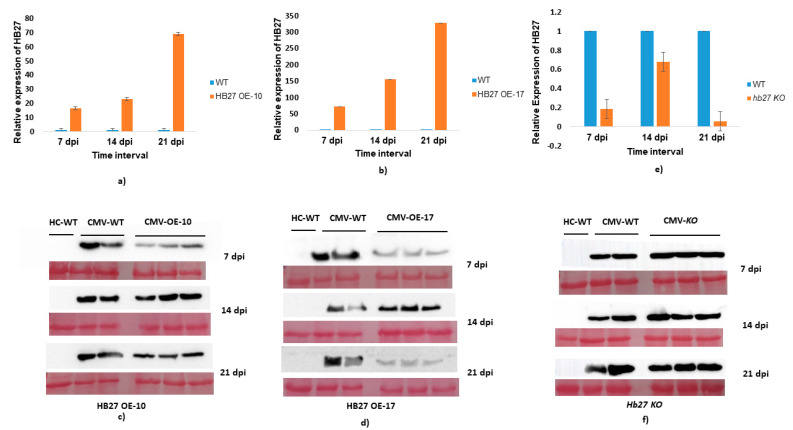
Determination of effects of HB27 overexpression and knockout on CMV. The transcript levels of HB27 were calculated in CMV-infected plants compared to healthy plants at 7, 14 and 21 dpi. The relative expression was estimated using the 2^−ΔΔCt^ method after the expression levels were normalized by 18S (**a**,**b**,**e**). CMV accumulation in overexpression and knockout was determined by Western blotting using coat protein-specific antiserum (**c**,**d**,**f**).

To analyze the effect of HB27 overexpression in CMV infection, two lines (OE-10 and OE-17) were chosen. In these lines, expression of HB27 was ~15- and ~45-fold higher, compared to wild type. Both lines were mechanically inoculated with CMV along with wild-type plants. In these HB27-OE lines, symptom development at 7 dpi is almost equivalent to wild-type plants but at later stages (14 dpi) symptom recovery was observed in HB27 OE lines compared to wild-type infected plants (Supplementary Figure S2). During infection, upregulation in HB27 in OE lines was observed. In line OE-10, HB27 expression was ~15-, 22- and 68-fold higher at 7, 14 and 21 dpi, respectively, (Figure 6a) as compared to wild-type plants. Similarly, in line OE-17 expression of HB27 was ~70-, 150- and 325-fold higher compared to wild-type plants (Figure 6b). The highest expression of HB27 in both lines was observed at 21 dpi. CMV accumulation in HB27-OE lines was determined using Western blotting (WB). In WB, less CMV accumulation was observed in line OE-10 on both 7 and 21 dpi (Figure 6c). Significantly less CMV coat protein accumulation was observed in line OE-17 on both 7 and 21 dpi, which can be related to the higher HB27 expression (Figure 6d). A decrease in virus accumulation reflects that overexpression of HB27 leads to the development of tolerance to CMV.

The effect of HB27 silencing upon CMV infection was studied using T-DNA insertion knockout lines (*hb27-KO*). CMV was mechanically inoculated onto *hb27-KO* and Col-0. When compared to wild-type plants and HB27 OE lines, symptom appearance in *hb27-KO* lines is early at 7 dpi, and it continuously spread in subsequent stages, such as 14 to 21 dpi (Supplementary Figure S2). When compared to wild-type plants, HB27 expression was 5.2-, 1.47- and 16- fold lower in the *hb27-KO* line (Figure 6e). CMV accumulation was substantially higher in *hb27-KO* lines than in wild-type plants, as evidenced using Western blotting (Figure 6f).

## 4. Discussion

TFs are known to play an important role in the development of plants. Studying the role of TFs during viral pathogenesis is essential for the elucidation of their role in plant defense. The homeobox TF family is known to regulate different functions during development and in plant defense against a broad range of pathogens [34,41,42]. POTH1, an important potato homeobox gene, regulates the development of leaf morphology and tuber numbers by acting as a negative regulator of GA biosynthesis [43]. In another study, the homologs of AtZHD1, i.e., BraZF-HD14, BraZF-HD25 and BraZF-HD28 were also found to be upregulated under biotic stress, such as ABA and PEG treatments [11]. In Arabidopsis, the overexpression of DREB1/CBF caused tolerance against abiotic stresses that include drought, freezing and salinity [44,45,46]. Tsip1, a zinc finger protein from *Nicotiana tabacum*, interacted with the 1a and 2a protein of CMV [47]. Overexpression of Tsip1 led to delays in symptom development and a reduction in CMV titer. Therefore, it is important to identify the host factors interacting with viral proteins and to understand the function of interaction, so that effective strategies can be developed for generations of virus resistance.

In the present study, we found that HB27, a homeodomain transcription factor from Arabidopsis, interacts with the CMV 2b protein. The interaction was confirmed using three techniques: yeast two-hybrid, far-Western blotting and BiFC. In BiFC, interaction was observed both in the nucleus and cytoplasm. CMV-2b contains two nuclear localization signal motifs [40] and these motifs are responsible for the localization of 2b to the nucleus. Apart from this, 2b is also localized in the cytoplasm [19]. Nuc-PLoc prediction analysis [48] showed that HB-27 is subnuclear localized. Therefore, based on these results, it can be presumed that the 2b protein interacts with HB-27 in the nucleus and alters its expression during pathogenesis. Further, it was observed that infection of CMV leads to ~3-fold upregulation of HB27 at 14 dpi, and minor downregulation at 21 dpi in Arabidopsis.

For functional characterization of the role of HB27-2b interaction, HB27 overexpressing and knockout lines were challenged with CMV and analyzed for CMV symptom development and accumulation. Lines overexpressing HB27 under the control of the 35S promoter were generated. Overexpression of HB27 was confirmed for both transcripts (using qRT-PCR) and protein (using Western blotting). Two different HB27 OE lines (HB27 OE-10 and HB27 OE-17) having different expression levels of the target gene were used. HB27 OE lines showed less accumulation of CMV at 7 and 21 dpi, and at 21 dpi showed recovery from disease symptoms compared to wild-type plants. HB27 is an essential host gene for CMV infection. Line 17 shows a high accumulation of HB27 at all analyzed times, especially at 21 dpi, and a significant reduction in virus titer and symptom recovery was observed in this line. However, in knockout lines, symptom severity and CMV accumulation were increased compared to wild type. Therefore, it can be concluded that HB27 sequesters 2b effectively in later infection stages (21 dpi). Members of the homeobox TF family are known to play important roles during biotic and abiotic stress. For example, HDTF1, a homeobox TF, was found downregulated during *Verticillium dahliae* and *Botrytis cinerea* infection of cotton. VIGS-mediated silencing of HDTF1 leads to the development of resistance against these pathogens [39]. Additionally, homeodomain-leucine zipper protein 1 is known to play an important role in CMV pathogenesis. Overexpression of homeodomain-leucine zipper protein 1 leads to increased disease severity, whereas its silencing leads to the development of disease tolerance by inducing defense-related genes [34]. The establishment of CMV infection depends on several host factors. As virus titer slowly rises in young tissue after establishing systemic infection (up to 14 dpi), the observed higher expression of HB27 at 14 dpi is triggered by this enhanced CMV titer. The resulting interaction between the host proteins (HB-27) and viral protein suppresses further spread, which is visible in lower virus accumulation at 21 dpi.

From these studies, it can be concluded that TFs play a critical role in biotic and abiotic stress responses and are prominent targets for engineering stress tolerance in transgenic plants. In consequence, it can be assumed that CMV 2b protein suppressed the expression of HB27, permitting the development of infection, and overexpression of this gene leads to the development of tolerance to CMV. Additionally, from BiFC results, it can be predicted that overexpression of HB27 effectively traps 2b in the nucleus, which leads to a decrease in its suppressor activity and generated tolerance by reducing CMV accumulation. This kind of phenomenon was also observed earlier with CMV and also with tomato bushy stunt virus P19 protein [18,49]. This study will lead to the identification of proteins regulated by homeobox, which leads to the development of tolerance. Modulation of these genes can lead to the development of virus-resistant/tolerant crops.

## Data Availability

The data presented in this study are available on request from the corresponding author.

## Supplementary Materials

The following are available online at https://www.mdpi.com/article/10.3390/pathogens11070788/s1, Figure S1: Far western blotting: Purified HB27-HA-pET28a and CMV2b-MYC-pET28a proteins were run along with BSA run on SDS-PAGE gel and stained with coomassie brilliant blue (a). the ponceau staining was done to ensure the transfer of proteins and consider as loading control (b). Figure S2: At 14 and 21 dpi, the symptoms of CMV on HB27-OE, hb27-KD, and wild type plants were compared to healthy wild type plants. When compared to HB27-KD and wild type plants, the severity of CMV infection in HB27-OE is lower. Table S1: List of primers used in this study. Underlined sequences represent the restriction sites.
