# Supplementary material for: Homeobox 27, a Homeodomain Transcription Factor, Confers Tolerances to CMV by Associating with Cucumber Mosaic Virus 2b Protein
Source: Pathogens. 2022 Jul 12;11(7):788. doi: 10.3390/pathogens11070788 (PMC9323240; doi:10.3390/pathogens11070788)
Supplement: Supplementary file 1 [file pathogens-11-00788-s001.zip › pathogens-1796017-supplementary.pdf]

Supplementary Figure S1:

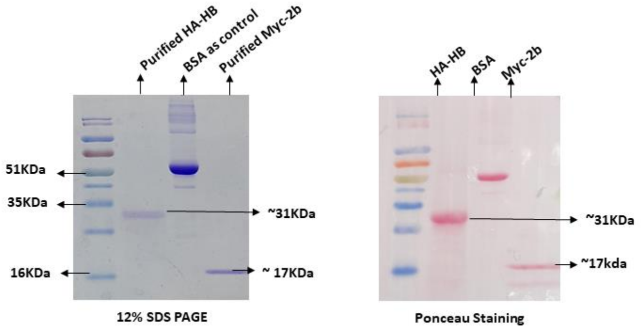

Far western blotting: Purified HB27-HA-pET28a and CMV2b-MYC-pET28a proteins were run along with BSA run on SDS-PAGE gel and stained with coomassie brilliant blue (a). the ponceau staining was done to ensure the transfer of proteins and consider as loading control (b).

Supplementary Figure S2:

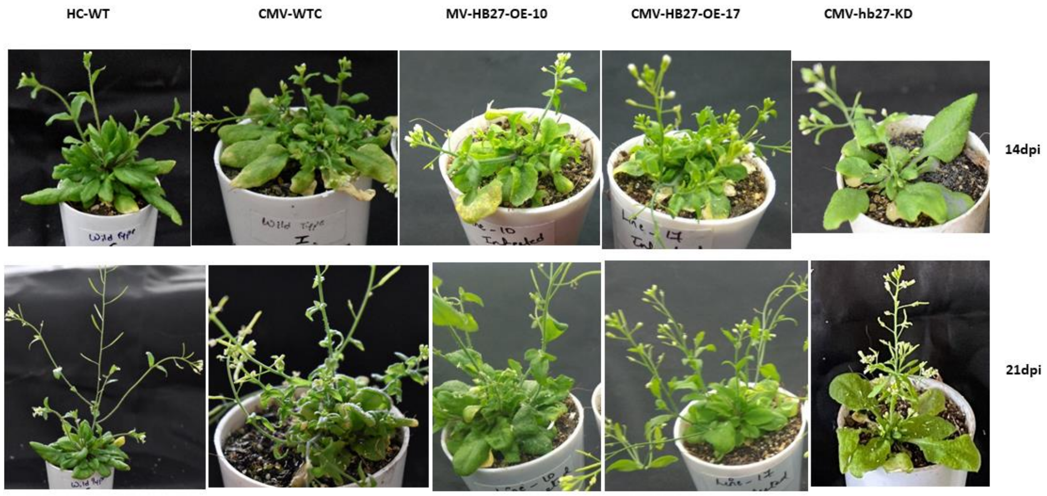

At 14 and 21 dpi, the symptoms of CMV on HB27-OE, hb27-KD, and wild type plants were compared to healthy wild type plants. When compared to HB27-KD and wild type plants, the severity of CMV infection in HB27-OE is lower.

Table S1: List of primers used in this study. Underlined sequences represent the restriction sites. Italicized sequences showed the HA and MYC taq sequences.

	**Y2H construct**
HB27	FP 5'AGATCTATGGATGAGATAAAACCAAA 3'<br>RP 5' CTCGAGTCATTCATCAATTATAATTT 3'
CMV 2b	FP 5' CATATGATGGATGTGTTGA 3'<br>RP 5' CCCGGGTCAAAACGACCCTTC 3'
	**BiFC construct**
HB27	FP 5' CTCGAGATGGATGAGATAAAACCAAAAC 3'<br>RP 5' ACTAGTTTCATCAATTATAATTTTATCC 3'
2b	FP 5' GGATCCATGGATGTGTTGACAGTAGTG 3'<br>RP 5' CTCGAGAAACGACCCTTCGGCCCATTCG 3'
	**Far western construct**
HB27	FP 5' GCAGCCATATG*TACCCATACGATGTTCCAGATTACGCT*GATGAGATAAAACCAAAG 3'<br>RP 5' TGGTGCTCGAGTCATTCATCAATTATAAT 3'
CMV 2b	FP 5' GGATCC*ATGGAACAAAAACTCATCTCAGAAGAGGATCTG*GATGTGTTGACAGTAG 3'<br>RP 5' TGGTGCTCGAGTTATCAAAACGACCCTTCG
	**Overexpression construct**
HB27	FP 5' AATAGATCTTATGGATGAGATAAAACCAAAG 3'<br>RP 5' TTAACTAGTTTCATCAATTATAATTTTATCC 3'
GFP	FP 5' GGAGTTGTCCCAATTCTTGTT 3'<br>RP 5' GTCTCTCTTTTCGTTGGGATC 3'
Hygromycin	FP 5' CATACAAGCCAACCACGGCCTCC 3'<br>RP 5' GCGTGGATATGTCCTGCGGGTA 3'
	**Mutant genotyping**
HB27 full length	FP 5' ATGGATGAGATAAAACCAAAGAAAGAAG 3'<br>RP 5' TTCATCAATTATAATTTTATCCTTGTG 3'
LBb1.3	5' ATTTTGCCGATTTCGGAAC 3'
	**Real time Primers**
18S	FP 5' TCTGCCCGTTGCTCTGATG 3'<br>RP 5' TCACCCGTCACCACCATAG 3'
HB27	FP 5' TCCTCGACTCAATCTTCAAA 3'<br>RP 5' TTCTCCGGGATTAACTCTTC 3'
CMV	FP 5' ACCCTGAAACCGCCTGAAAT 3'<br>RP 5' TCCGAACTGTAACCCACACG 3'
